# A Single-Center Prospective Study on Adverse Drug Reactions Associated With Polypharmacy in Elderly Outpatients

**DOI:** 10.7759/cureus.98532

**Published:** 2025-12-05

**Authors:** Maria Khurshid, Ammarah Amjad, Sundas Qamar, Muhammad Amir, Adnan Khan, Muhammad Iftikhar Khattak, Muhammad Rizwan Umer, Tahir Iqbal Mirza

**Affiliations:** 1 Pharmacology, Azad Jammu and Kashmir Medical College, Muzaffarabad, PAK; 2 Pharmacology, HBS Medical and Dental College, Rawalpindi, PAK; 3 Geriatrics, Russell’s Hall Hospital, Dudley, GBR; 4 Medicine, Yangtze University, Yangtze, CHN; 5 Gastrointestinal Surgery, Yangtze University, Yangtze, CHN; 6 Research and Development, Health Services Academy, Islamabad, PAK; 7 Trauma Surgery, Royal Sussex County Hospital, Brighton, GBR; 8 Surgery, Combined Military Hospital, Kharian, PAK

**Keywords:** adverse drug reactions, comorbidities, elderly, naranjo algorithm, outpatient care, polypharmacy

## Abstract

Background: Polypharmacy in elderly patients significantly increases the risk of adverse drug reactions (ADRs), posing a challenge to safe outpatient care.

Objective: To prospectively evaluate the frequency, nature, and clinical outcomes of ADRs associated with polypharmacy in elderly outpatients.

Methodology: A prospective observational study was conducted at the outpatient department of Azad Jammu and Kashmir Medical College (AJKMC), Muzaffarabad, over one year from June 2023 to May 2024. Through convenience sampling, 246 individuals who were at least 60 years old and using five or more drugs were included. Medical record reviews and structured patient interviews were used to gather data. The FDA Toxicity Grading Scale was used to classify ADRs according to their severity after they were evaluated using the FDA Toxicity Grading Scale. SPSS version 25.0 (IBM Corp., Armonk, NY) was used for the statistical analysis, and *P* < 0.05 was chosen as the significance level.

Results: Of the 246 patients, 132 patients (53.66%) were male and 114 patients (46.34%) were female. The most common comorbidities were hypertension in 151 patients (61.38%), type 2 diabetes mellitus in 103 patients (41.87%), and ischemic heart disease in 89 patients (36.18%). Regarding polypharmacy, 94 patients (38.21%) were taking 5-6 medications, 87 patients (35.37%) were on 7-8 medications, and 65 patients (26.42%) were on 9 or more medications. A total of 76 patients (30.89%) experienced at least one ADR. The most frequently affected systems were gastrointestinal in 28 patients (11.38%) and dermatological in 17 patients (6.91%). ADRs were significantly more common in patients aged ≥80 years (16 out of 41, 39.02%), in those taking ≥9 medications (30 out of 65, 46.15%), and in those with two or more comorbidities (52 out of 121, 43.70%) (*P* < 0.05 for all comparisons).

Conclusions: Polypharmacy in elderly outpatients is strongly associated with clinically significant ADRs, warranting routine medication review and vigilant pharmacovigilance.

## Introduction

The older population is rapidly expanding due to the worldwide increase in life expectancy, which has made age-related health issues a higher emphasis in clinical practice [1]. Elderly people are more susceptible to chronic diseases and often need to take multiple drugs to control their conditions [2]. Polypharmacy, or the simultaneous use of numerous drugs, has therefore become a major concern in geriatric medicine [3]. Although often necessary to manage comorbidities, polypharmacy substantially increases the risk of adverse drug reactions (ADRs), drug-drug interactions, and poor medication adherence [4,5]. These problems not only threaten patient safety but also increase morbidity, mortality, healthcare utilization, and reduce quality of life [5].

Physiological changes associated with aging further complicate drug therapy. Alterations in pharmacokinetics and pharmacodynamics, such as reduced renal and hepatic clearance, altered metabolism, and increased drug sensitivity, can diminish therapeutic benefit while amplifying toxicity [6]. In addition, cognitive decline, sensory impairments, and low health literacy may hinder adherence to complex regimens, increasing the likelihood of ADRs [7].

The risk is particularly high in outpatient settings where elderly patients generally self-administer medications without close monitoring [8]. The concomitant use of over-the-counter products, duplicate prescriptions from multiple providers, and lack of comprehensive medication reviews further elevate the chance of harmful drug events [9]. Despite these risks, ADRs among older outpatients are often underreported, especially in low- and middle-income countries (LMICs) with limited pharmacovigilance infrastructure [10,11].

Recent studies underscore the growing prevalence of polypharmacy in Asia. More than 45% of elderly outpatients were reported to be on five or more medications, with nearly one-third exposed to potentially inappropriate drugs [12]. In Pakistan, community-based surveys have shown that polypharmacy affects nearly 30-40% of older adults, with a significant proportion experiencing preventable ADRs [13]. Similarly, studies from China, Japan, and Ethiopia highlight increasing rates of medication-related harm in older populations, emphasizing the urgent need for region-specific data and interventions [14,15,16].

Given these gaps, there is a critical need for prospective evaluations that document the incidence, types, and clinical outcomes of ADRs associated with polypharmacy in elderly outpatients, particularly within LMIC healthcare systems where monitoring is limited. The objective of this study was to prospectively evaluate the frequency, nature, and clinical consequences of ADRs in elderly outpatients exposed to polypharmacy in a single-center setting.

## Materials and methods

Study design and setting

This prospective observational study was conducted in the outpatient department of Azad Jammu and Kashmir Medical College (AJKMC), Muzaffarabad, from June 2023 to May 2024 to identify and analyze adverse drug reactions (ADRs) among elderly outpatients with polypharmacy.

Inclusion and exclusion criteria

Patients aged 60 years or older who attended the outpatient department during the study period and were taking five or more medicines simultaneously were eligible. Only patients providing verbal or written consent were enrolled. Patients were excluded if they lacked adequate medication records, were hospitalized at the time of data collection, or had severe cognitive impairment as defined by a Mini-Mental State Examination (MMSE) score ≤ 17, which precluded reliable participation without a caregiver. Patients receiving end-of-life or palliative care were also excluded. While these criteria were necessary for feasibility and data accuracy, we acknowledge that such exclusions may bias results toward healthier elderly populations and could influence the observed ADR prevalence.

Sample size

Using a convenience sampling method, 246 elderly outpatients were enrolled. The use of convenience sampling reflected the single-center setting and the intention to include all eligible patients presenting during the study period. No a priori power calculation was performed, as the study was exploratory in nature; however, the achieved sample size aligns with similar observational studies in comparable settings [12,13,14,15,16]. We recognize that convenience sampling may introduce selection bias and that larger, multi-center studies with formal sample size estimation would strengthen generalizability.

Data collection

Data were collected through structured face-to-face interviews complemented by detailed prescription and medical record reviews. To minimize recall bias, patients were encouraged to bring their medication strips, containers, or written records to appointments. Interviews were conducted using a standardized questionnaire developed for this study, with all research staff trained uniformly to reduce interviewer variability. Collected variables included demographics, comorbidities, medication profiles, and ADR characteristics.

ADR causality was assessed using the WHO-UMC Causality Assessment System [17], and severity was classified using the FDA Toxicity Grading Scale [18]. To enhance reliability, two independent clinical pharmacologists evaluated ADRs, with discrepancies resolved through consensus discussion. We acknowledge that some subjectivity remains, as confirmatory laboratory testing was not always feasible. The full interview tool is provided for transparency in the Appendix.

Statistical analysis

Data were analyzed using SPSS version 25.0 (IBM Corp., Armonk, NY). Descriptive statistics (means, standard deviations, frequencies, and percentages) were used to summarize patient characteristics and ADR profiles. Chi-square tests were employed to explore associations between ADR incidence and variables such as age, gender, number of prescriptions, and comorbidities, with *P* < 0.05 considered significant. We acknowledge that the absence of multivariate adjustment may have left residual confounding unaccounted for, and future studies should employ regression modeling to address this limitation.

Ethical approval

The Institutional Ethical Review Committee of AJKMC examined and approved the research protocol. Before recruitment, all patients gave their informed permission, and patient confidentiality and anonymity were rigorously maintained throughout the research.

## Results

Table 1 outlines the age, gender, and comorbidities of 246 elderly outpatients. Most patients were aged 60-69 years (112, 45.53%), followed by 70-79 years (93, 37.80%), and ≥80 years (41, 16.67%). Males slightly outnumbered females (132, 53.66%, vs. 114, 46.34%). The most common comorbidities were hypertension (151, 61.38%), type 2 diabetes (103, 41.87%), and ischemic heart disease (89, 36.18%), with lesser prevalence of chronic kidney disease (47, 19.11%), chronic obstructive pulmonary disease (COPD)/asthma (33, 13.41%), osteoarthritis (28, 11.38%), and depression/anxiety (22, 8.94%).

**Table 1:** Demographic and clinical characteristics of the study population (n = 246). COPD, chronic obstructive pulmonary disease

Category	Variable	Frequency (*n*)	Percentage (%)
Age group (years)	60-69	112	45.53
	70-79	93	37.80
	≥80	41	16.67
Gender	Male	132	53.66
	Female	114	46.34
Comorbidities	Hypertension	151	61.38
	Type 2 diabetes mellitus	103	41.87
	Ischemic heart disease	89	36.18
	Chronic kidney disease	47	19.11
	COPD/Asthma	33	13.41
	Osteoarthritis	28	11.38
	Depression/Anxiety	22	8.94

**Figure 1:**
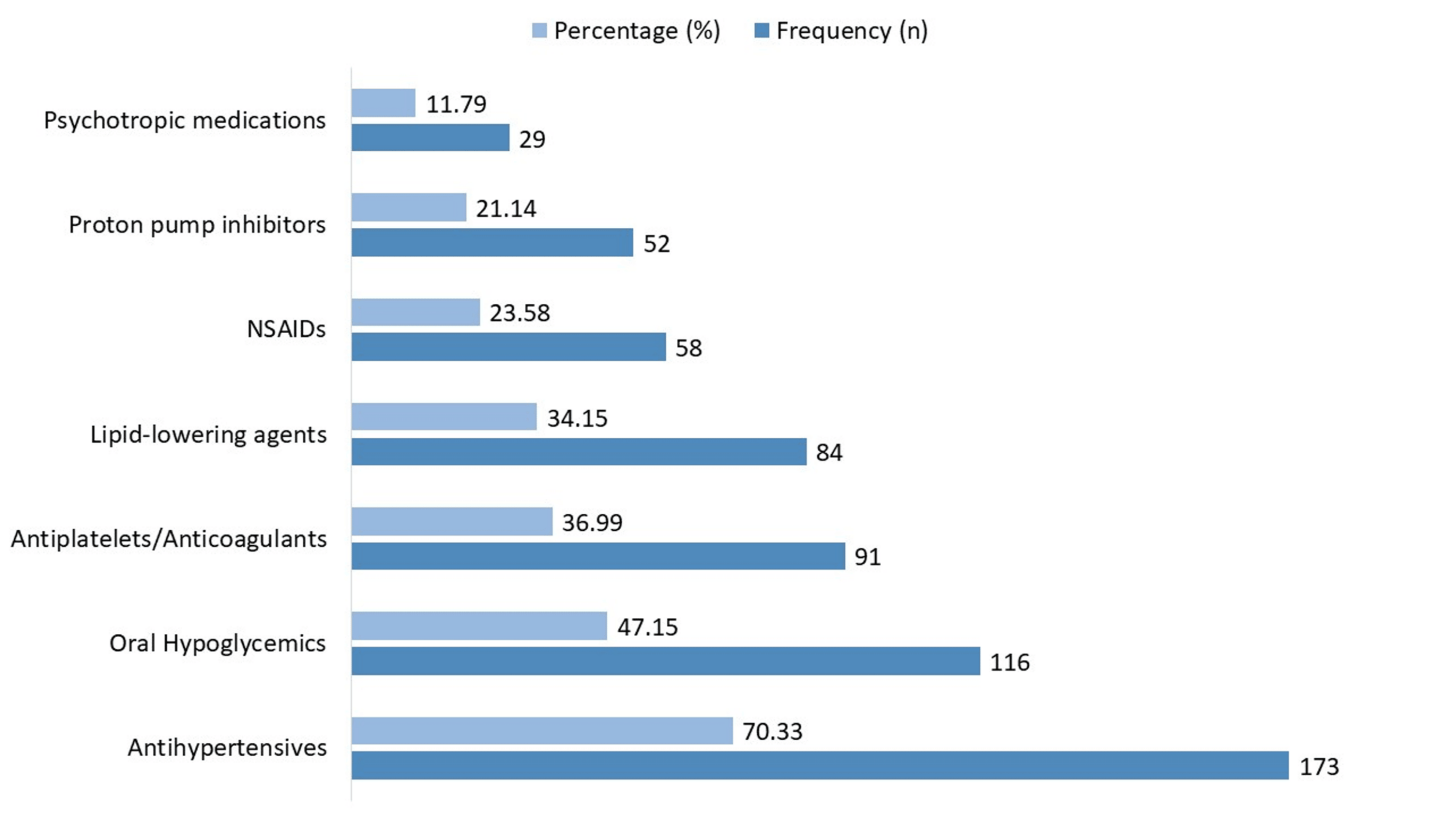
Common therapeutic classes of medications used. NSAIDs, nonsteroidal anti-inflammatory drugs

Among the elderly outpatients, the most frequently used medication classes were antihypertensives (173 patients, 70.33%), followed by oral hypoglycemics (116, 47.15%), antiplatelets/anticoagulants (91, 36.99%), and lipid-lowering agents (84, 34.15%) (Figure 1). Nonsteroidal anti-inflammatory drugs (NSAIDs) were used by 58 patients (23.58%), proton pump inhibitors by 52 patients (21.14%), and psychotropic medications by 29 patients (11.79%).

**Figure 2:**
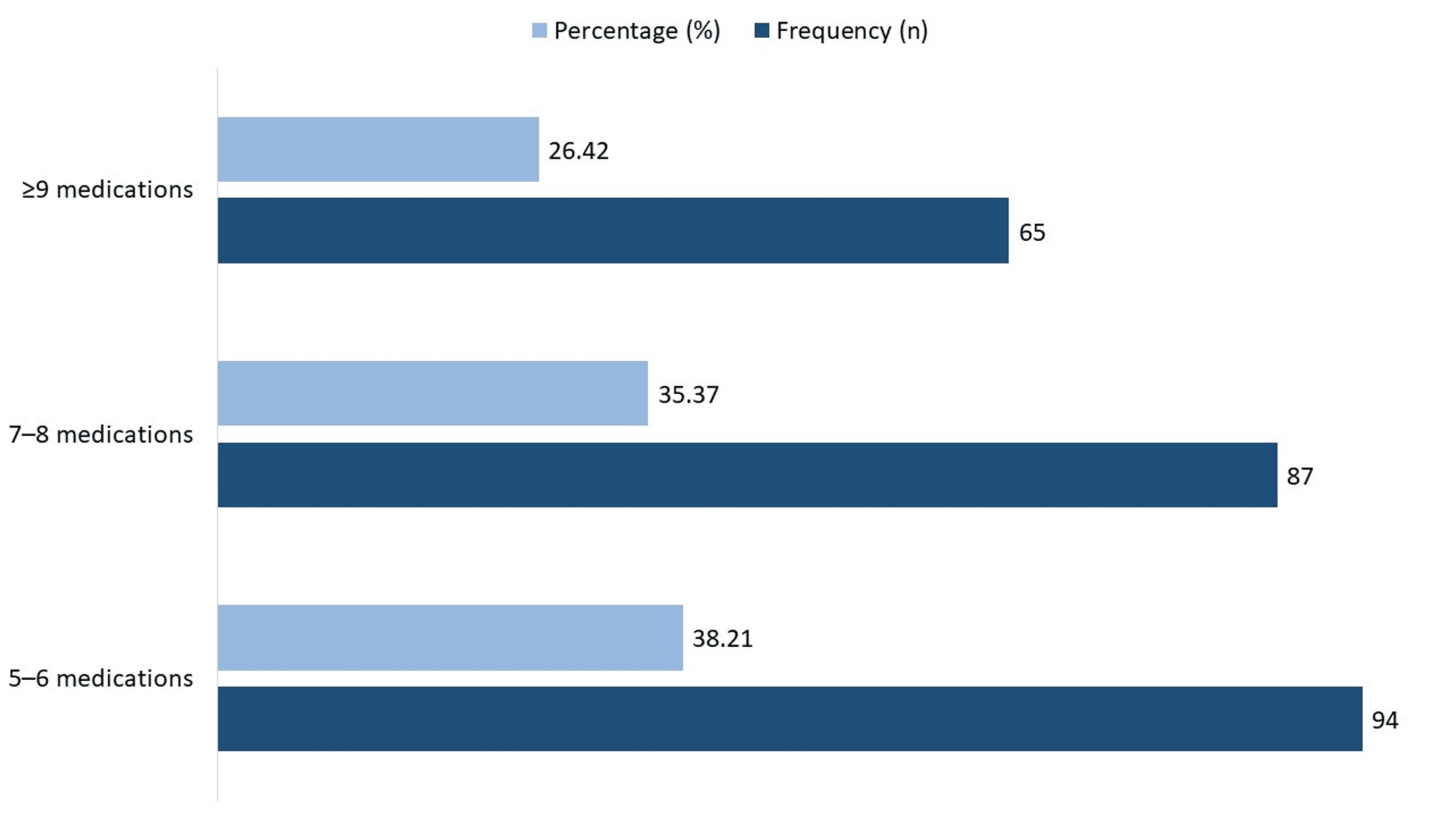
Number of medications taken by patients.

Figure 2 displays the distribution of polypharmacy levels. Among 246 patients, 94 (38.21%) were on 5-6 medications, 87 (35.37%) on 7-8 medications, and 65 (26.42%) were taking 9 or more medications concurrently.

Out of 246 patients, 76 (30.89%) experienced at least one adverse drug reaction (ADR), while 170 (69.11%) reported none (Table 2). The most common ADRs were gastrointestinal (28, 11.38%), followed by dermatological (17, 6.91%), neurological (12, 4.88%), cardiovascular (10, 4.07%), and others such as fatigue or insomnia (9, 3.66%).

**Table 2:** Prevalence and categorized characteristics of reported ADRs (n = 246). *Percentages calculated out of 76 patients with ADRs. ADR, adverse drug reaction; FDA, U.S. Food and Drug Administration

Category	ADR characteristic	Frequency (*n*)	Percentage (%)
ADR prevalence	Patients with ≥1 ADR	76	30.89
	Patients without ADRs	170	69.11
Type of ADR (system affected)	Gastrointestinal (nausea, diarrhea)	28	11.38
	Dermatological (rash, itching)	17	6.91
	Neurological (dizziness, confusion)	12	4.88
	Cardiovascular (hypotension, bradycardia)	10	4.07
	Others (e.g., fatigue, insomnia)	9	3.66
Severity (FDA Toxicity Grading Scale)	Mild	31	40.79*
	Moderate	36	47.37*
	Severe	9	11.84*
Clinical outcomes	ER visit required	12	15.79*
	Hospitalization required	7	9.21*
	Managed in an outpatient clinic	57	75.00*
ADR reporting	Reported to the national ADR center/WHO-UMC	19	25.00*
	Not reported	57	75.00*

**Figure 3:**
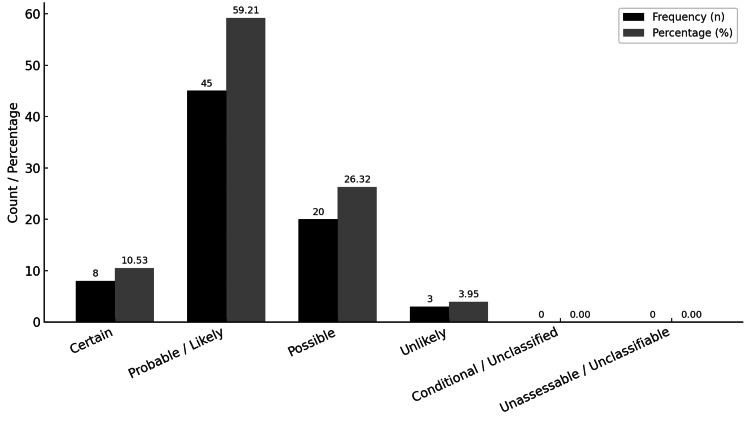
Distribution of adverse drug reaction (ADR) cases according to the WHO-UMC system for standardized case causality assessment. WHO-UMC, World Health Organization-Uppsala Monitoring Centre

Among the 76 patients with ADRs, causality was classified as probable in 45 cases (59.21%), possible in 20 (26.32%), definite in 8 (10.53%), and doubtful in 3 (3.95%) using the FDA Toxicity Grading Scale (Figure 3).

**Figure 4:**
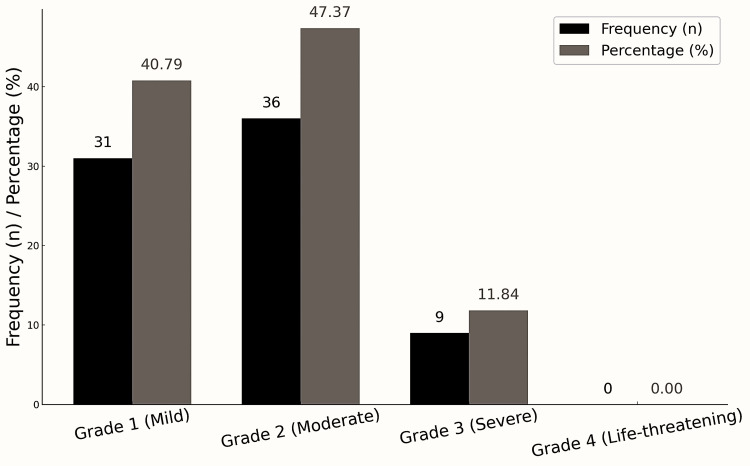
Severity of ADRs (FDA Toxicity Grading Scale, n = 76). ADR, adverse drug reaction; FDA, U.S. Food and Drug Administration

In terms of severity, 36 ADRs (47.37%) were moderate, 31 (40.79%) were mild, and 9 (11.84%) were classified as severe, indicating that the majority of ADRs were non-life-threatening but still clinically significant (Figure 4).

Statistical analysis showed significant associations between ADR occurrence and both age and polypharmacy (Table *3*). ADRs were more common in patients aged ≥80 (16, 39.02%) and those on ≥9 medications (30, 46.15%). Patients with ≥2 comorbidities had a higher ADR rate (52, 42.97%) compared to those with fewer (24, 19.20%). No significant gender difference was found (*P* = 0.582).

**Table 3:** Association between ADR occurrence and patient variables (n = 246). A *P*-value < 0.05 was considered statistically significant; multivariate logistic regression adjusted for age, gender, comorbidity burden, and polypharmacy. ADRs, adverse drug reactions; χ², chi-square statistic; CI, confidence interval

Category	Variable	Patients with ADRs (*n*, %)	Patients without ADRs (*n*, %)	Total (*n*)	*P*-value (χ²-test)	Adjusted odds ratio (95% CI)*	*P*-value (logistic regression)
Age group	60-69 years	28 (25.00%)	84 (75.00%)	112	0.041	Reference	-
	70-79 years	32 (34.41%)	61 (65.59%)	93		1.42 (0.78-2.61)	0.244
	≥80 years	16 (39.02%)	25 (60.98%)	41		1.89 (0.87-4.09)	0.106
Gender	Male	38 (28.79%)	94 (71.21%)	132	0.582	Reference	-
	Female	38 (33.33%)	76 (66.67%)	114		1.12 (0.64-1.97)	0.682
Polypharmacy	5-6 medications	18 (19.15%)	76 (80.85%)	94	0.001	Reference	-
	7-8 medications	28 (32.18%)	59 (67.82%)	87		1.68 (0.85-3.31)	0.132
	≥9 medications	30 (46.15%)	35 (53.85%)	65		2.75 (1.34-5.64)	0.006
Comorbidity burden	<2 comorbidities	24 (19.20%)	101 (80.80%)	125	0.003	Reference	-
	≥2 comorbidities	52 (42.97%)	69 (57.02%)	121		2.34 (1.25-4.37)	0.008

## Discussion

This prospective investigation highlighted the clinical susceptibility of older outpatients exposed to polypharmacy by finding a significant prevalence of ADRs (76, 30.89%). Compared to those reported in similar conditions, the observed ADR rate is much greater. For instance, prior research at a tertiary care hospital in Karachi reported an ADR incidence of 10.5% among elderly patients receiving polypharmacy [19]. Our cohort’s higher frequency may be attributable to differences in patient characteristics, prescribing practices, healthcare system dynamics, or the stricter ADR detection and reporting procedures employed in our study.

Our results also revealed clear age-related patterns in ADR prevalence. ADR incidence was highest in patients aged ≥80 years (16, 39.02%), followed by those aged 70-79 years (32, 34.41%) and 60-69 years (19, 25.00%) (*P* = 0.041). These findings align with prior research linking advanced age with heightened drug sensitivity and pharmacokinetic changes that increase susceptibility in the oldest-old group [20].

Polypharmacy significantly influenced ADR occurrence. ADRs were reported in 30 (46.15%) patients taking ≥9 medications, 28 (32.18%) of those prescribed 7-8 medications, and 18 (19.15%) of those taking 5-6 medications (*P* = 0.001). This dose-response trend reinforces polypharmacy as a major risk factor, consistent with earlier studies where patients experiencing ADRs used significantly more medications on average (10.5 vs. 7.8) [21].

Comorbidity burden also played a crucial role. ADRs were reported in 52 of 121 patients (43.70%) with ≥2 chronic illnesses, compared with 24 patients (19.20%) with fewer comorbidities (*P* = 0.003). This is in line with previous literature suggesting that multimorbidity exacerbates pharmacological complexity, thereby elevating ADR risk [22].

The most frequent ADRs involved the gastrointestinal system (28, 11.38%), followed by dermatological (17, 6.91%) and neurological (12, 4.88%) manifestations. These trends are similar to those reported in elderly ambulatory cohorts where gastrointestinal and neurological ADRs predominate [23]. According to the WHO-UMC causality assessment, 45 ADRs (59.21%) were classified as probable/likely, and 20 ADRs (26.32%) as possible. Importantly, while no ADR-related deaths were recorded, many were clinically significant, with 18.42% requiring emergency room visits or unscheduled physician consultations, and 6.57% necessitating short-term hospitalization. All identified ADRs were reported to the national pharmacovigilance program under the Drug Regulatory Authority of Pakistan (DRAP), ensuring compliance with WHO global monitoring standards.

Strengths and limitations

One of this study’s major strengths is its prospective design, which facilitated systematic and real-time identification of ADRs using validated instruments such as the FDA Toxicity Grading Scale and the WHO-UMC system for standardized causality assessment. By focusing on an elderly outpatient population in a resource-limited setting, our findings contribute novel, region-specific evidence to the global polypharmacy literature. Furthermore, outcome-level data on healthcare utilization provide clinical context beyond incidence rates, underscoring the tangible burden of ADRs on patients and the health system.

However, some limitations must be acknowledged. The single-center design restricts generalizability, and convenience sampling may introduce selection bias. Although real-time data collection minimized recall bias, underreporting of mild, self-limiting symptoms remains possible. Causality assessments were primarily clinical and not universally supported by laboratory confirmation. Additionally, the absence of a control group and long-term follow-up precluded evaluation of delayed-onset ADRs. Future multi-center studies with longitudinal designs are needed to validate these findings, incorporate more robust causality confirmation, and further examine healthcare utilization and economic impact.

## Conclusions

This research shows that polypharmacy is strongly linked to a higher incidence of ADRs in older outpatients, especially those who are 80 or older, use 9 or more drugs, or have more than one chronic illness. The gastrointestinal and skin systems were the most impacted, and most ADRs were of moderate severity and likely to be the cause. These results show how important it is to regularly assess medications, increase pharmacovigilance, and tailor prescriptions to each patient in order to make medications safer for older persons in outpatient care settings.

## Appendices

Appendix 

**Table 4:** Structured Interview Questionnaire.

Section 1: Demographics	
Patient ID (anonymous code)	_______
Age	_______ (Years)
Gender	☐ Male ☐ Female
Marital Status	☐ Single ☐ Married ☐ Widowed ☐ Divorced
Education Level	☐ Illiterate ☐ Primary ☐ Secondary ☐ Graduate ☐ Other: _______
Section 2: Medical History	
Comorbidities (tick all that apply)	☐ Hypertension ☐ Diabetes ☐ Ischemic Heart Disease ☐ Chronic Kidney Disease ☐ COPD/Asthma ☐ Osteoarthritis ☐ Depression/Anxiety ☐ Other: _______
Number of comorbidities	☐ <2 ☐ ≥2
Family history of chronic illness	☐ Yes ☐ No
Section 3: Medication History	
Total number of prescribed medications	_______
Number of medications taken concurrently	☐ 5–6 ☐ 7–8 ☐ ≥9
Duration of medication use	_______ years / months
List of current medications	__________________________
Use of OTC/herbal medications	☐ Yes ☐ No
Therapeutic Classes (tick all that apply)	☐ Antihypertensives ☐ Oral hypoglycemics ☐ Antiplatelets/Anticoagulants ☐ Lipid-lowering agents ☐ NSAIDs ☐ Proton Pump Inhibitors ☐ Psychotropic drugs ☐ Others: __________
Section 4: Adverse Drug Reactions (ADRs)	
Experienced new/unusual symptoms?	☐ Yes ☐ No
If yes, describe	__________________________
System involved	☐ Gastrointestinal ☐ Dermatological ☐ Neurological ☐ Cardiovascular ☐ Other: __________
Onset of symptoms	__________
Action taken	☐ Stopped drug ☐ Dose changed ☐ Medical visit ☐ Hospitalization ☐ Other: __________
Outcome of ADR	☐ Resolved ☐ Ongoing ☐ Recurrence ☐ Serious
Causality (WHO-UMC system)	☐ Certain ☐ Probable / Likely ☐ Possible ☐ Unlikely ☐ Conditional / Unclassified ☐ Unassessable / Unclassifiable
Severity (FDA Toxicity Grading Scale)	☐ Grade 1 (Mild) ☐ Grade 2 (Moderate) ☐ Grade 3 (Severe) ☐ Grade 4 (Life-threatening)
Section 5: Adherence and Monitoring	
Do you take all medications as prescribed?	☐ Yes ☐ No
Do you sometimes forget to take your medications?	☐ Yes ☐ No
Do you use pill organizers/reminders?	☐ Yes ☐ No
Do you have a caregiver to help with medication management?	☐ Yes ☐ No
Do you have regular medication review by a physician?	☐ Yes ☐ No
Frequency of follow-up visits	☐ Monthly ☐ Every 3 months ☐ Every 6 months ☐ Yearly
